# Predictors of Cognitive Impairment in Cancer Survivors: Using the Korean Longitudinal Study of Aging (KLoSA)

**DOI:** 10.1093/geroni/igaf122.3206

**Published:** 2025-12-31

**Authors:** Ah Rim Lee, Kyeongin Cha, Xirong Cui, Jeongeun Hwang, Sun Suk Bhin, Dahyeon Lee, Misook Jung

**Affiliations:** Chungnam National University, Daejeon, Republic of Korea; Chungnam National University, Daejeon, Republic of Korea; Chungnam National University, Daejeon, Republic of Korea; Chungnam National University, Daejeon, Republic of Korea; Chungnam National University, Daejeon, Republic of Korea; Chungnam National University, Daejeon, Republic of Korea; Chungnam National University, Daejeon, Republic of Korea

## Abstract

Aging populations and increasing numbers of older cancer survivors demand targeted cognitive health management. Cancer diagnosis and treatment may acccelerate cognitive aging, leading to faster decline than normal aging. Advanced statistical methods are crucial to unravel the complex factors influencing post-treatment cognition. Accordingly, this study employed latent growth mixture models to delineate heterogeneous cognitive trajectories, followed by the development of machine learning model to predict key factors influencing cognitive changes among cancer survivors. Data from 313 cancer survivors, aged from 55 to 95 years, were extracted from a cohort of 6,940 older individuals who participated in the 1st and 7th waves of the Korean Longitudinal Study of Aging. The analysis included demographic information, health-related behaviors, dementia-related comorbidities, cancer-related factors, and cognitive screening scores. Two cognitive trajectories emerged: a maintenance group exhibiting stable, normal scores (84.0%) and a decline group with decreasing scores (16.0%). A broad set of predictors was used in the predictive models to classify membership in the identified cognitive trajectories. The machine learning models-logistic regression, random forest, artificial neural network, support vector machine-achieved balanced accuracies ranging from 0.84 to 0.86 and AUC values between 0.74 and 0.88. Key predictors included age, difficulty with daily activities due to cancer, depression, time since diagnosis, and social networks. These findings underscore the heterogeneous nature of cognitive aging in older cancer survivors and both cancer-related and dementia-related factors play critical roles in predicting cognitive decline. Comprehensive, personalized interventions to mitigate the multifactorial influences on cognition are needed in this vulnerable population.

